# Results of an Integrated Phase I/II Prospective Clinical Trial (NEXIS) for Neoadjuvant Anti-PD-L1 (Durvalumab) and Anti-CTLA-4 (Tremelimumab) With Radiation for High-Risk Soft-Tissue Sarcoma of the Trunk and Extremities

**DOI:** 10.7759/cureus.72119

**Published:** 2024-10-22

**Authors:** Vincent Y Ng, Mario N Sahlani, Jessa D Fogel, Anthony K Chiu, Michael E Kallen, Derik Davis, James Snider, William Regine, Søren M Bentzen, Edward Sausville

**Affiliations:** 1 Department of Orthopedics, University of Maryland Medical Center, Baltimore, USA; 2 Department of Pathology, University of Maryland School of Medicine, Baltimore, USA; 3 Department of Diagnostic Radiology, University of Maryland Medical Center, Baltimore, USA; 4 Department of Radiation Oncology, University of Maryland School of Medicine, Baltimore, USA; 5 Greenebaum Comprehensive Cancer Center and Department of Epidemiology and Public Health, University of Maryland School of Medicine, Baltimore, USA; 6 Greenebaum Comprehensive Cancer Center, University of Maryland School of Medicine, Baltimore, USA

**Keywords:** cancer immunotherapy, neoadjuvant radiation therapy, pd-l1 inhibitors, percist, recist, soft-tissue sarcoma

## Abstract

Background

The current management of large, high-grade soft tissue sarcoma (STS) of the trunk and extremities includes radiation and surgical resection. The initial use of chemotherapy and targeted therapy are controversial and although most patients present with localized disease, many eventually develop incurable metastases. The results and analysis of the safety and antitumor activity of combined checkpoint inhibitor immunotherapy with neoadjuvant radiation for high-risk primary STS are presented here.

Methods

This was an integrated phase I/II prospective single-arm trial (Nutrition and Exercise in Critical Illness Trial (NEXIS) trial). Eligible patients were age ≥18 years with histologically confirmed intermediate or high-grade STS of the trunk or extremity ≥5 cm diameter and were Eastern Cooperative Oncology Group performance status 0-1. The treatment algorithm included neoadjuvant anti-PD-L1 (Durvalumab) and anti-CTLA-4 (Tremelimumab) for three cycles of four weeks/cycle along with external beam radiation for five weeks, followed by wide surgical resection, and adjuvant Durvalumab monotherapy for four cycles. High-grade toxicity was continually assessed for the first 12 patients in phase I and the primary endpoint for phase II was an excellent histological response (grade 0 or 1 score on a semi-quantitative assessment for tumor regression). This study was registered with ClinicalTrials.gov, number NCT03116529.

Findings

Between October 2017 and November 2021, 23 patients were enrolled. Five patients had progression of distant disease during neoadjuvant treatment and withdrew from the study before surgery. A total of 18 patients who completed at least the neoadjuvant immunotherapy, radiation and surgery were included for analysis. The most common tumor was undifferentiated pleomorphic sarcoma (n=9, 50%). The occurrence of any adverse event (AE) was recorded in 16 (88.9%) patients, and 3 (16.7%) patients had a serious AE. Eight out of 18 patients (44.4%) had disease-free survival at a median of 39.7 months. Four out of 18 patients (22.2%) were alive-with-disease at a median of 37.1 months from diagnosis of distant metastasis, and six out of 18 (33.3%) died of disease at a median of 20.8 months from diagnosis of distant metastasis. Local recurrence occurred in two patients (11.1%) and was concomitant with distant disease in each case. Based on Response Evaluation Criteria in Solid Tumors v1.1, a partial response was noted in five (27.8%) cases, stable disease in 10 (55.6%) cases, and progressive disease in three (16.7%) cases. Histological semiquantitative analysis revealed a "good" response in eight (44.4%) patients, a "moderate" response in four (22.2%) patients, and a "poor" response in six (33.3%) patients. The mean patient-reported outcome measures regarding fatigue, physical function, or physical interference demonstrated no significant differences between various timepoints before, during, or after treatment.

Conclusion

Neoadjuvant combined immunotherapy and radiation for high-risk STS was relatively well-tolerated. The histological, radiologic, and clinical outcome data in this novel trial were relatively similar to historical literature for non-immunotherapy treatment regimens.

## Introduction

Soft tissue sarcoma (STS) accounts for approximately 12,000 new cases and 5,000 deaths annually in the United States [1]. Sixty-five percent of sarcomas are grade 2 (intermediate/IG) or 3 (high/HG) and have a five-year overall survival (OS) of 75% and 45-55%, respectively [2]. Although there are different subtypes of STS, size and histological grade are the most important prognostic factors [3]. Less than 10% of STS are associated with clinically detectable metastases at presentation, but nearly 50% of all high-risk STS will develop distant metastasis. The vast majority (>80%) of metastases occur within two years of definitive treatment. Without treatment or after cessation of treatment, the median survival of patients with metastatic STS is approximately six months, and with treatment, median survival ranges from nine to 15 months [1,3]. At two years, approximately 30-40% of patients who develop metastases will remain alive-with-disease [4].

The current standard of care for high-risk STS (Grade 2/3, size greater than or equal to 5 cm, located deep or superficial to fascia) of the pelvis and extremities is neoadjuvant or adjuvant radiation therapy (RT) and wide surgical resection [5]. This results in local control rates greater than 85-90% and isolated local recurrence rarely leads to mortality [5]. Metastatic STS, however, is generally considered incurable, with only approximately 5-15% of patients alive at five years depending on the timing and extent of distant relapse [3,5]. The role of initial cytotoxic chemotherapy is highly controversial and varies widely among centers [3]. Because the vast majority of high-risk STS present with localized disease yet a high proportion develop incurable metastatic disease, there has been considerable effort over the past 40 years to develop effective regimens to prevent the development of distant disease. Unfortunately, there have been limited improvements in survival with either chemotherapy or targeted therapy [3,5]. The need for novel approaches to prevent distant relapse of STS is clear.

Cancers are recognized by the immune system and, under some circumstances, the immune system may control or even eliminate tumors. Manipulation of co-stimulatory or co-inhibitory signals can amplify T-cell responses against tumors. This amplification may be accomplished by blocking co-inhibitory receptors such as cytotoxic T‑lymphocyte-associated antigen 4 (CTLA-4) and programmed cell death 1 (PD‑1) from binding with their ligands, B7 and programmed cell death ligands 1 and 2 (PD-L1, PD-L2), respectively. Targeting both PD-1 and CTLA-4 pathways may have additive or synergistic activity because the mechanisms of action of CTLA-4 and PD-1 are non-redundant [6]. Preclinical data has shown that approximately 60% of STS express PD-1/PD-L1 and its presence was associated with disease progression [7]. In a series of 29 cases of chondrosarcoma and liposarcoma, PD-L1 on tumor cells was found to be present in 100% of cases, but only 11% and 45% were found to have PD-1 positive infiltrating T-cells [8]. Increased CTLA-4 T-cells were found in aggressive pediatric sarcoma patients and the mutagenic burden of tumors has been correlated with the strength of tumor-specific immune response with checkpoint inhibitor therapy [9].

Multiple clinical studies and small animal models have demonstrated superior rates of response for checkpoint blockade when combined with RT or with another checkpoint receptor compared to immunotherapy alone [9,10]. Neoadjuvant or adjuvant radiation therapy combined with wide surgical resection has been shown to improve local control of the primary tumor compared to surgery alone. While the effects of radiation on tumor necrosis have long been reported, the effects on the immune system have been the focus of more recent interest [11]. Ionizing radiation reverses the immunosuppressive tumor microenvironment, facilitates tumor-antigen recognition by cytotoxic T-lymphocytes, increases tumor expression of PD-L1 and leads to increased immune activity against local and distant tumor cells [10,12-17]. In addition to the immunogenic effects of RT on the primary tumor, the well-described abscopal effect of localized RT on distant metastases is widely felt to be immune-mediated. Numerous small animal tumor models and multiple clinical case examples have been reported where localized RT had a beneficial effect on other sites of disease, and that the use of immunotherapy and RT are often synergistic in achieving an abscopal effect [18-26]. When combined with RT, anti-PD-1 has been shown to strongly potentiate both the local and the abscopal effect of RT, leading to primary tumor regression and resistance to the implantation of secondary or metastatic tumors [10,27]. In this manner, RT may facilitate utilization of the primary tumor essentially as an in-situ vaccine to train the immune system and eliminate or prevent metastases, even in weakly immunogenic tumors [28].

There have been clinical trials for checkpoint inhibitors as a second-line agent for patients with advanced or unresectable sarcoma. In contradistinction, this investigator (VYN)-initiated trial (Nutrition and Exercise in Critical Illness Trial (NEXIS)) proposed using an anti-PD-L1 agent, Durvalumab (AstraZeneca, Cambridge, England) and an anti-CLTA-4 agent, Tremelimumab (AstraZeneca) as a front-line combination treatment in the neoadjuvant setting with RT for an enhanced abscopal effect to address micro- or oligo-metastatic disease when the immune system has not been yet depleted by chemotherapy or massive tumor burden. The rationale for this study was to add an effective checkpoint inhibitor combination to the existing standard of care (RT plus surgery) to potentiate the immunomodulatory effect of both modalities, thereby overcoming the limited systemic effect seen in prior studies with single-pathway blockade, RT-alone, or those with low mutagenic burden tumor phenotypes. Furthermore, based on the timing of immunotherapy, the treatment algorithm would not delay the completion of the standard of care with radiation and surgery. Harnessing these synergistic immunomodulatory effects could suggest a promising novel approach to the treatment of high-risk STS. The hypotheses of this study were that this treatment would be relatively well tolerated by patients, demonstrate a favorable histologic response in the primary tumor, and support further studies to define whether improvement of oncologic outcomes results from this approach.

## Materials and methods

Study design and participants

Patients with high-risk trunk or extremity-based histologically proven STS were screened for enrollment. High-risk STS were defined as tumors greater than or equal to 5 cm, intermediate or high grade according to FNCLCC (Federation Nationale des Centres de Lutte Contre le Cancer) criteria and a location deep or superficial to fascia. Intermediate or high-grade tumors that were locally recurrent, metastatic, or had prior inadequate resections were also included as high risk. Histologies included the most common adult-type STS such as undifferentiated pleomorphic sarcoma, liposarcoma, leiomyosarcoma, fibrosarcoma, synovial sarcoma, angiosarcoma, epithelioid sarcoma and malignant peripheral nerve sheath tumor. Histologies that were primarily bone-based sarcomas that can occur in the soft tissues (for example, extraskeletal Ewing sarcoma) and predominantly low-grade STS (for example, solitary fibrous tumor, well-differentiated liposarcoma, dermatofibrosarcoma protuberans) were excluded. Retroperitoneal STS, surgically unresectable primary tumors, and patients with more than two liver metastases, more than two soft tissue, non-lymph node (for example, intramuscular, subcutaneous) metastases, or liver or soft tissue non-lymph node metastases >1 cm were excluded. Patients were ≥18 years old, had ECOG (Eastern Cooperative Oncology Group) performance status ≤1 and had adequate organ and marrow function. Patients who have had prior cytotoxic chemotherapy or targeted systemic therapy (for example, Pazopanib, Trabectidin) were included as long as it was greater than four weeks prior to the start of immunotherapy. Patients who received any prior checkpoint inhibitor treatment, had immunosuppression, had immunodeficiency or autoimmune conditions, had active infection, or had inflammatory bowel disease were excluded. 

The protocol and all amendments were approved by the Institutional Review Board (study #HM-HP-00073356-25). The study was performed in accordance with the principles of the Declaration of Helsinki and the International Conference on Harmonization Good Clinical Practice guidelines and overseen by a steering committee. All patients provided written, informed consent for participation. All patients were discussed at a multi-disciplinary tumor board. This study was registered with ClinicalTrials.gov, number NCT03116529. This study was supported by funds through the Maryland Department of Health’s Cigarette Restitution Fund Program (CH-649-CRF) and the National Cancer Institute Cancer Center Support Grant (CCSG) P30CA134274. The immunotherapy drugs were provided by the manufacturer, AstraZeneca.

Interventions

**Figure 1:**
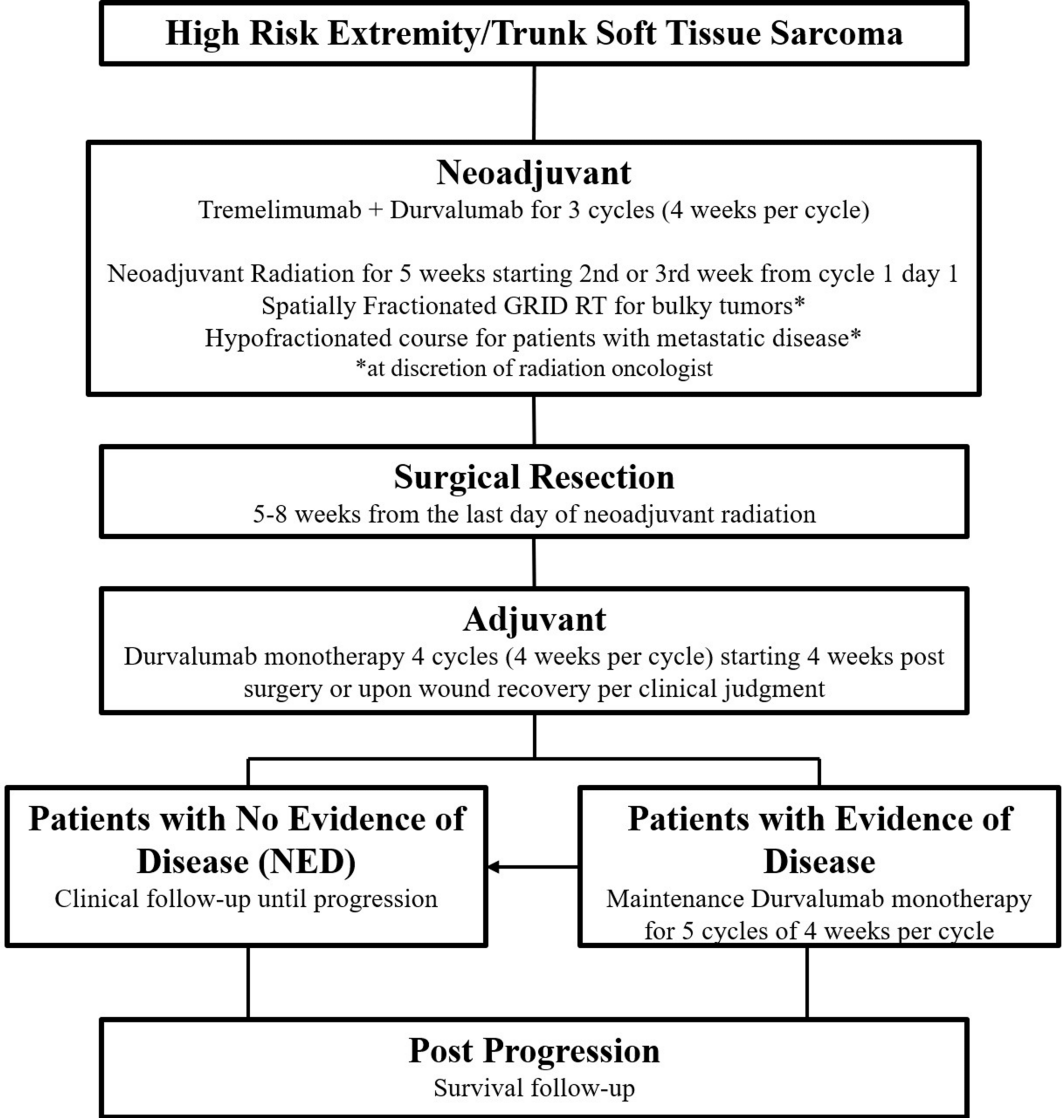
Treatment Algorithm

Immunotherapy was administered according to the algorithm in Figure 1. Fixed dosing of Durvalumab 1500 mg intravenous (IV) and Tremelimumab 75 mg IV for patients > 30 kg and weight-based dosing of Durvalumab 20 mg/kg q4w and Tremelimumab 1 mg/kg q4w for patients < 30 kg were utilized. For radiation, patients received external beam radiation therapy (EBRT) to the primary tumor and a margin of normal tissue with a minimum dose of 50 Gy and 1.8-2 Gy per fraction. For bulky sarcomas, defined as > 10 cm in greatest dimension, a single 15 Gy fraction of spatially fractionated GRID radiation therapy (SFGRT) was delivered to the gross tumor volume within one to three days prior to the start of EBRT. For surgery, en-bloc removal of the primary tumor with negative margins of ≥2 cm distance (muscle/fat) or of anatomic boundaries (fascia/periosteum) was endeavored depending on the proximity of vital structures. If any unplanned positive margins after primary resection were noted, a re-resection of margins was performed if possible.

Assessments

Safety assessments were evaluated based on the CTCAE v4.0 (Common Terminology for Adverse Events) and RTOG Toxicity Criteria (Radiation Therapy Oncology Group). Patient pain, fatigue and function were assessed at baseline, after neoadjuvant treatment, after surgery, and after adjuvant monotherapy using the Patient Reported Outcomes Measurement Information System (PROMIS) - PROMIS-CA Bank v1.1 Physical Function, PROMIS Pain Interference SF8a v1.0 and PROMIS Pain Interference SF6b v1.0 questionnaires - validated functional measures created by the National Institutes of Health (NIH).

A magnetic resonance imaging (MRI) of the primary tumor, a dedicated computed tomography scan (CT) of the chest, abdomen, and pelvis, and a whole-body positron-emission tomography scan (PET-CT) was performed before and after neoadjuvant treatment, and an MRI of the primary site and CT chest was performed for surveillance after surgery every three months for the first two years, every six months until year 5, and then annually until year 10. The Response Evaluation Criteria in Solid Tumors (RECIST) v1.1 was used to classify the treatment response of the primary lesion and any metastatic involvement and the duration of response. PET response was determined by changes in peak standardized uptake value (SUVpeak) from baseline to follow-up in accordance with the modified PET Response Criteria in Solid Tumors (PERCIST 1.0).

For histologic treatment response in STS, no current consensus exists. All cases were analyzed using a semiquantitative assessment for tumor response based upon the Modified Ryan Scheme for Tumor Regression that takes into account measures of residual viable tumor, coagulative necrosis, hyalinization/fibrosis and infarction [29]. In addition, treatment response was graded based on the five-tier, stainable tumor cell-based, EORTC-STBSG response score (European Organization for Research and Treatment of Cancer-Soft Tissue and Bone Sarcoma Group) [30].

Oncologic outcomes were recorded including local recurrence (LR), distant relapse (DR), overall survival (OS) and disease-specific survival (DSS) for all patients, disease-free survival (DFS) for patients presenting with localized disease, and progression-free survival (PFS) for patients presenting with metastases. Survival was measured from the time of surgical resection. 

Statistical methods and data analysis

The phase I component established the safety and provided a provisional characterization of the toxicity profile of D/T/RT by continual assessment of high-grade toxicity in the first 12 cases enrolled for the trial. The phase I component used a Pocock boundary to make a decision to stop or continue the trial after each patient was evaluated with respect to early toxicity. Staggered entry of cases was allowed and for patient safety reasons, no more than four patients were allowed to be on active neoadjuvant treatment at any point during the phase I portion of the trial. All patients enrolled during the phase I component were evaluable with respect to efficacy as well. A full analysis of toxicity and efficacy, including clinical efficacy endpoints, was conducted. 

The phase II component used a Simon two-stage design with good or excellent response (grade 0 or 1 score on a semi-quantitative assessment for tumor regression) on histopathologic examination as the efficacy endpoint for the phase II component. Based on the literature review for cytotoxic chemotherapy, it was proposed for this study that if an excellent response was lower than 20%, the combination was not superior to existing modalities in this regard, whereas a response rate of 40% would be seen as promising for further development [31]. A sample size of 33 patients was established as the enrollment target such that 18 patients would be accrued in the first stage of Simon’s two-stage design and if greater than four excellent responses were noted, an additional 15 patients would be accrued.

Data was organized in Microsoft Excel (Microsoft Corp., Redmond, WA, USA). Patient demographics, tumor characteristics, adverse events, and outcomes of interest were reported as frequencies and proportions in terms of percentages. For comparison of PROMIS scores at progressive time points, the paired student’s T-test was used for normally distributed samples and the paired Wilcoxon’s rank-sum test was used as the non-parametric alternative where applicable. The Shapiro-Wilk test was used to assess normality. An a priori alpha was set at 0.05 for the interpretation of significance. All statistical operations were performed using R software (R Core Team 2023. R: A Language and Environment for Statistical Computing. R Foundation for Statistical Computing, Vienna, Austria).

## Results

Demographics

Data was analyzed for patients who completed the complete course of neoadjuvant immunotherapy and underwent surgical resection of the primary lesion. Of the 23 patients who began the study, four patients had progression of the distant disease before completion of the neoadjuvant treatment and one patient with localized disease had unmanageable discomfort and disability at the primary site due to the size of the primary tumor. The study was aborted in these five patients at the discretion of the treating team and patient. A total of 18 patients were included in the analysis. Although the primary efficacy endpoint for phase II was achieved after accruing the target of 18 patients for the first stage of Simon two-stage design, the trial was discontinued early due to slower than expected accrual. Demographics are listed in Table 1.

**Table 1:** Patient Demographics *Prior to completion of neoadjuvant radiation, immunotherapy, and surgery

Demographic Characteristic	n (%)
Participants Enrolled	
Enrolled	23
Included in Analysis	18 (78.3%)
Withdrew From Study*	5 (21.7%)
Age at Diagnosis (Average Years, 95% CI)	52.2 [20.4 - 84.0]
Males	11 (61.1%)
Females	7 (38.9%)
Race	
White	14 (77.8%)
Black or African American	3 (16.7%)
Asian	1 (5.6%)
Ethnicity	
Hispanic or Latino	2 (11.1%)
Primary Tumors	16 (88.9%)
Recurrent Tumors	2 (11.1%)
Original Site of Primary Tumor	
Thigh	11 (61.1%)
Lower Leg	4 (22.2%)
Elbow	1 (5.6%)
Groin	1 (5.6%)
Thigh, Hip	1 (5.6%)
Histology	
Myxofibrosarcoma	2 (11.1%)
Myxoid / Round Cell Liposarcoma	4 (22.2%)
Myxoid Spindle Cell	1 (5.6%)
Synovial Sarcoma	1 (5.6%)
Undifferentiated Pleomorphic Sarcoma (UPS)	9 (50.0%)
Unclassifiable	1 (5.6%)
Received Adjuvant Therapy	
Any Adjuvant Therapy	13 (72.2%)
Adjuvant Durvalumab Monotherapy Only	6 (33.3%)
Adjuvant Chemotherapy or Radiation Only	5 (27.8%)
Adjuvant Durvalumab + Chemotherapy or Radiation	2 (11.1%)

Safety (phase I)

A total of 16 patients (88.9%) experienced one or more adverse events during their course of treatment. The most common adverse events were fatigue (n=5, 27.8%), rash (n=5, 27.8%), and thyroid dysfunction (n=4, 22.2%). Three patients (16.7%) had a combined total of five serious adverse events. One patient was diagnosed with bilateral pulmonary emboli four weeks after completing the first cycle of neoadjuvant immunotherapy. This patient also had postoperative hemorrhage after surgical resection. A second patient had a wound infection leading to sepsis 16 days after surgical resection. A third patient developed immune-mediated colitis after the third cycle of neoadjuvant immunotherapy and after receiving the COVID-19 (coronavirus disease of 2019) immunization. The COVID-19 immunization, however, may have been unrelated, as it has been reported to not significantly increase the risk of immune-mediated side effects of immunotherapy [32]. The adverse effects can be found in Table 2.

**Table 2:** Safety, Adverse Events in As-Treated Population All adverse events reported were deemed to be possibly due to immunotherapy regimen. Reported as number of patients who experienced each event. *Asterisk used to denote serious adverse events

Adverse Event (AE)	n (%)
Serious AE^*^	3 (16.7%)
Any AE	16 (88.9%)
Fatigue	5 (27.8%)
Rash	5 (27.8%)
Anorexia	3 (16.7%)
Nausea	3 (16.7%)
Pruritus	3 (16.7%)
Diarrhea	2 (11.1%)
Fever	2 (11.1%)
Hyperthyroidism	2 (11.1%)
Hypothyroidism	2 (11.1%)
Weight loss	2 (11.1%)
Abdominal pain	1 (5.6%)
Delayed orgasm	1 (5.6%)
Granulomas	1 (5.6%)
Hyponatremia	1 (5.6%)
Immune related colitis^*^	1 (5.6%)
Increased appetite	1 (5.6%)
Injection site reaction	1 (5.6%)
Leg swelling	1 (5.6%)
Lymphopenia	1 (5.6%)
Mucositis oral	1 (5.6%)
Palmar-plantar erythrodysesthesia syndrome	1 (5.6%)
Post-operative hemorrhage^*^	1 (5.6%)
Prosthetic joint infection^*^	1 (5.6%)
Pulmonary embolism^*^	1 (5.6%)
Skin fungal infection	1 (5.6%)
Somnolence	1 (5.6%)
Testicular pain	1 (5.6%)
Thrombus	1 (5.6%)
Wound infection^*^	1 (5.6%)

Efficacy (phase II)

At presentation, of the 18 patients that completed neoadjuvant immunotherapy and radiation, none had definite metastases although two patients (11.1%) had hypermetabolic lymph nodes: one with hypermetabolic axillary lymph nodes and one with hypermetabolic external iliac and femoral lymph nodes (Table 3). After completion of the neoadjuvant treatment, a total of four patients (22.2%) were reported to have metastases on restaging imaging. Six additional patients (33.3%) later developed metastases after surgery, with an average of 20 months postop (range 2-43 months). Two patients (11.1%) had local recurrence after surgery, which was concomitant with distant metastases. Of the 10 patients (55.6%) with distant metastases, four (22.2%) were alive with disease and six (33.3%) died of disease at latest follow-up (Table 4). Of note, one patient (5.6%), a 29-year-old male who had a 14 cm undifferentiated pleomorphic sarcoma (UPS) and had slowly enlarging multiple bilateral lung metastases first detected two months after surgery, remained alive-with-disease at latest follow-up (nearly 70 months) despite having no systemic therapy, pulmonary metastatectomy, or radiation to the lung nodules.

For the particular STS subtypes of UPS, myxofibrosarcoma and myxoid/round cell liposarcoma, the rates of patients who remained disease-free were 33% (3/9), 100% (2/2), and 50% (2/4). Two of UPS patients and two of the myxoid/round cell liposarcoma patients were diagnosed with metastases after neoadjuvant treatment but before surgery. 

**Table 3:** Disease Progression in Study Participants *Went on to develop metastases **Concomitant with distant metastases ~Mean ± standard deviation, median and range in brackets ^From date of surgical resection

Stage of Treatment, Metric of Disease Progression	n (%)
At Presentation:	
Total Patients	18
Metastases	0 (0%)
Hypermetabolic Nodes	
Axillary Nodes	1 (5.6%)
External Iliac and Femoral Nodes^*^	1 (5.6%)
Diagnosed after Immunotherapy, Before Surgery:	
Metastases	
Soft Tissue	1 (5.6%)
Soft Tissue, Lung, and Abdominal	1 (5.6%)
Bone and Lung	1 (5.6%)
Liver	1 (5.6%)
Diagnosed after Surgery:	
Metastases	
Lung	5 (27.8%)
Lung and Salivary Gland	1 (5.6%)
Local Recurrence**	2 (11.1%)
End of Study:	
Overall Survival	12 (66.7%)
Mean Follow-Up (Months) ^	44.2 ± 18.3~
Median Follow-Up (Months) ^	47.4 [3.0-71.7]
Disease-Free Survival	8 (44.4%)
Mean Follow-Up (Months) ^	37.9 ± 18.4~
Median Follow-Up (Months) ^	39.7 [3.0-63.2]

**Table 4:** Survival of Patients After Diagnosis of Distant Metastasis *Survival and follow-up measured from CT diagnosis of metastasis **Range

Total Patients Who Developed Metastatic Disease	10 (55.6%)
Survival Rate*	
3 months	10 (100%)
6months	9 (90%)
1 year	7 (70%)
2 years	5 (50%)
3 years	4 (40%)
Overall	4 (40%)
Patients who Died of Disease	6 (60%)
Median Survival (months)*	20.8 (4.3-49.9)**
Patients Alive with Disease at Latest Follow-Up	4 (40%)
Median Follow-Up (months)*	37.1 (18.5-69.7)**

**Figure 2:**
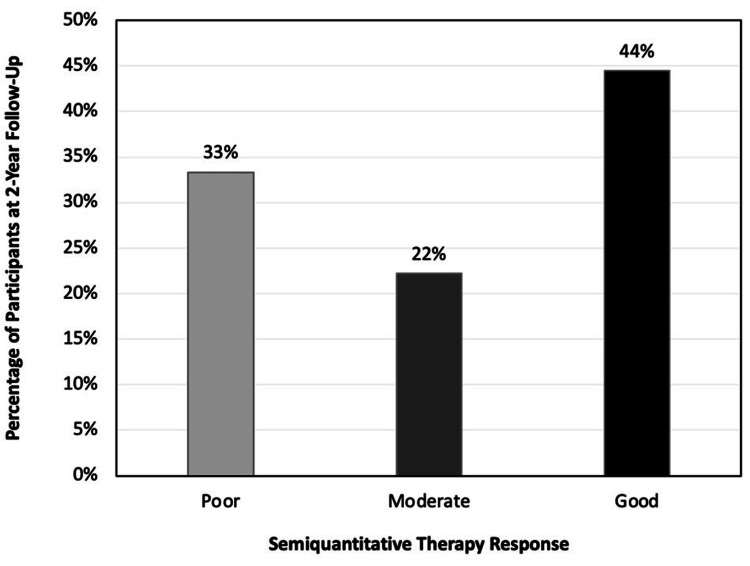
Percentage of patients and semiquantitative tumor specimen analysis

None of the patients had a complete response based on RECIST criteria; four patients (22.2%) were found to have a complete response with PERCIST criteria. The largest number of patients (n=8, 44%) had stable disease with RECIST criteria and partial response (n=6, 33.3%) with PERCIST criteria. Progressive disease was noted with RECIST criteria in three patients (16.7%) and with PERCIST criteria in one patient (5.6%). No patient was given an excellent rating on the semiquantitative analysis of the tumor specimen. There were histologically stainable tumor cells present in all the samples. These results can be found in Table 5 and Figure 2.

**Table 5:** Efficacy Outcomes EORTC-STBSG, European Organization for Research and Treatment of Cancer-Soft Tissue and Bone Sarcoma Group; RECIST, Response Evaluation Criteria in Solid Tumours; PERCIST, Positron Emission Tomography Response Criteria in Solid Tumors

Metric of Therapeutic Response	Definition	n (%)
RECIST v1.1		
Complete Response (CR)	Disappearance of target lesion	0 (0.0%)
Partial Response (PR)	At least a 30% decrease in target lesion	5 (27.8%)
Stable Disease (SD)	Neither sufficient shrinkage to qualify for PR nor sufficient increase to qualify for PD	10 (55.6%)
Progressive Disease (PD)	At least a 20% increase in the diameter of target lesion and must also demonstrate an absolute increase of at least 5 mm	3 (16.7%)
PERCIST		
Complete Response (CR)	No uptake	4 (22.2%)
Partial Response (PR)	≥30% decrease in uptake	7 (38.9%)
Stable Disease (SD)	In between	4 (22.2%)
Progressive Disease (PD)	≥30% increase in uptake or new hot lesions	1 (5.6%)
Data Unavailable		2 (11.1%)
Semiquantitative Analysis		
Excellent	No viable cancer cells (complete response)	0 (0%)
Good	Single cells or rare small groups of cancer cells (near complete response)	8 (44.4%)
Moderate	Residual cancer with evident tumor regression, but more than single cells or rare small groups of cancer cells (partial response)	4 (22.2%)
Poor	Extensive residual cancer with no evident tumor regression (poor or no response)	6 (33.3%)
EORTC-STBSG		
A	No stainable tumor cells	0 (0%)
B	Single stainable tumor cells or small clusters (overall below 1% of the whole specimen)	3 (16.7%)
C	1% to 10% stainable tumor cells	1 (5.6%)
D	10% to 50% stainable tumor cells	7 (38.9%)
E	Greater than 50% stainable tumor cells	7 (38.9%)

Patient-reported outcome measures

There were no significant differences in the patient-reported outcome measures regarding fatigue, physical function, and physical interference as indicated by PROMIS scores as patients progressed through treatment (Table 5).

**Table 6:** Patient-Reported Outcome Measures PROMIS: Patient Reported Outcomes Measurement Information System *Paired t-test or paired Wilcoxon rank sum test as the non-parametric alternative where applicable

PROMIS Time Point	Number of Responses	Average Score	95% CI	p-value*
Short Form 13a- Fatigue				
Baseline	18	22.3	7.9 - 36.7	
Pre-Surgery	16	24.2	9.2 - 39.2	0.498
Before Adjuvant Monotherapy	5	24.8	10.2 - 39.4	0.625
End of Treatment/After Adjuvant Monotherapy	7	22.4	6.8 - 38	0.836
Short Form 20a- Physical Function				
Baseline	18	77.1	55.8 - 98.4	
Pre-Surgery	14	76.9	49.2 - 104.6	0.289
Before Adjuvant Monotherapy	6	77	56 - 98	0.516
End of Treatment/After Adjuvant Monotherapy	8	72.2	43.5 - 100.9	0.353
Short Form 8a- Physical Interference				
Baseline	17	17.8	0.6 - 35	
Pre-Surgery	13	19.2	4.6 - 33.8	0.812
Before Adjuvant Monotherapy	7	16.6	0 - 33.9	0.604
End of Treatment/After Adjuvant Monotherapy	6	12.2	2.8 - 21.6	0.086

## Discussion

In the NEXIS trial, neoadjuvant combination anti-PD-L1/anti-CTLA-4 immunotherapy administered with radiation in patients with high-risk STS was associated with rates of 44% (n=8) good histological response, 28% (n=5) partial response for RECIST (though no complete response for RECIST), and 22% (n=4) complete response for PERCIST. At a median follow-up of nearly 40 months, 44% (n=8) of patients were alive with no evidence of disease. Of the patients who developed distant disease, 40% (n=4) of them remained alive-with-disease at 37 months after diagnosis of metastasis. No new safety concerns were identified in this regimen.

The rates of disease-free survival and the survival of patients with distant disease in this trial were similar to published data for STS treatment algorithms using radiation, surgery with or without chemotherapy [1-4]. It is possible that different doses, combinations, or types of immunotherapy treatments are necessary to benefit from the synergistic effects of RT and immunotherapy. Furthermore, while not performed in this study, it may be beneficial to conduct future studies assessing for tumor expression of PD-1/PD-L1/PD-L2, as this may correlate with more significant response to anti-PD-L1 treatment or predict superior results with anti-PD-L2 agents or anti-PD-1 agents that inhibit both PD-L1 and PD-L2. Since the initiation of NEXIS in 2017, other clinical trials examining checkpoint inhibitors for STS have also been performed. They are summarized below.

The vast majority of the existing immunotherapy clinical trials for STS have focused on metastatic or unresectable disease. Based on a systematic review, the median progression-free survival with immunotherapy for these patients ranged from 1.4 to over 11.2 months and the median overall survival from 5.6 to 34.7 [9]. Certain subtypes such as alveolar soft-part sarcoma or UPS in some cases demonstrated greater response rates.

Nivolumab 

In the setting of advanced or recurrent STS, none of the 21 patients responded to nivolumab (anti-PD-1) monotherapy in a phase 2 trial [33]. When paired with trabectedin, nivolumab demonstrated a 23.5% response rate and median progression-free survival (PFS) of 11.6 months in patients with metastatic sarcoma (18 of 20 were STS) [34]. This was favorable compared to trabectedin alone which a prior study had shown a median PFS of only 4.2 months [35]. These two studies, however, were not directly comparable since the latter was limited to liposarcoma and leiomyosarcoma whereas the former included UPS and other subtypes. A more recent study published in 2021 examined nivolumab with trabectedin as a second-line treatment in advanced, non-L (liposarcoma, leiomyosarcoma) STS patients. The six-month progression-free survival rate at six months was 23.1% and 8.7% for patients treated with early (cycle 2) and late (cycle 4) addition of nivolumab to trabectedin, respectively. The median duration of disease stabilization for all patients was four months [36]. Another phase II study examined the combination of sunitinib, a tyrosine kinase receptor inhibitor, and nivolumab in 58 evaluable patients with advanced or metastatic STS. The progression-free survival at six months was 48%. In 21% of the patients, an objective response was recorded and the 18-month overall survival for these patients was 100% [37].

Pembrolizumab

Pembrolizumab (anti-PD-1) monotherapy demonstrated an objective response in 18% of the patients with metastatic or locally unresectable STS and who had received at least three lines of prior systemic treatment. The highest rates were seen in UPS (40%) and liposarcoma (20%). The median progression-free survival was 18 weeks, although it was 30 weeks for patients with UPS [38]. Pembrolizumab was also examined in rare and ultra-rare sarcomas that were advanced stage or refractory to other treatment. With extended follow-up greater than 12 weeks, 17.5% of patients had an objective response. The best responses were seen in alveolar soft part sarcoma with the longest median progression-free survival of 6.6 months. Nevertheless, there was significant heterogeneity between different subtypes and the median PFS of the group as a whole was only 2.8 months [39]. The French Sarcoma Group examined the efficacy of Pembrolizumab combined with metronomic cyclophosphamide in 57 patients with unresectable leiomyosarcoma, UPS, other sarcomas and gastrointestinal stromal tumors. A partial response was seen in only one case (solitary fibrous tumor) and the six-month non-progression rate was 0% for leiomyosarcoma and UPS [40].

Ipilimumab

In a pilot study, ipilimumab (anti-CTLA-4) monotherapy was ineffective in six patients with advanced or metastatic synovial sarcoma. All patients showed clinical or radiologic evidence of disease progression after three or lower cycles of ipilimumab and the study was terminated early [41]. The Alliance A091491 study randomized 85 patients with advanced or metastatic sarcoma to nivolumab alone or nivolumab with ipilimumab; an objective response was seen in 5% and 16%, respectively [42]. Expansion cohorts found that the combination of nivolumab and ipilimumab led to a 14% 6-month response rate in UPS and dedifferentiated liposarcoma [43].

Neoadjuvant immunotherapy

There is limited existing data on checkpoint inhibitors in the neoadjuvant setting for STS. A small study randomized extremity/truncal UPS to neoadjuvant radiation plus nivolumab (anti-PD-1) or ipilimumab plus nivolumab. Out of nine UPS patients, two patients had disease relapse and one had progressive metastatic disease on treatment at a minimum of two years follow-up. Similar findings were reported with nivolumab alone and ipilimumab with nivolumab. No association was noted between progression-free survival and histologic tumor response or RECIST response [44,45]. Very recent results have been released for the clinical trial SU2C-SARC032 that compared neoadjuvant radiation and pembrolizumab followed by surgery and adjuvant pembrolizumab to neoadjuvant radiation and surgery for pleomorphic/dedifferentiated liposarcoma and UPS including myxofibrosarcoma of the extremity. They reported a superior 2-year DFS of 70% for the experimental group compared to 53% for standard of care, but no currently statistically significant difference in local recurrence free survival, distant DFS, or OS [46].

Study limitations

This study had several limitations. Recruiting patients proved difficult. Over the five-year enrollment period, 23 patients were recruited, but five (21.7%) withdrew due to not completing the neoadjuvant course. Only two study sites recruited patients and one site only yielded a single patient. Although difficulties exist, future studies should aim to enroll more recruitment sites. The heterogeneity of tumor subtypes proved difficult for sub-group analysis. This could also be remedied by increasing enrollment.

## Conclusions

The data presented here on the prospective clinical trial (NEXIS) demonstrated that neoadjuvant combination immunotherapy with radiation for high-risk STS was relatively well-tolerated by patients. No definitive radiologic, pathologic or clinical breakthroughs compared to the existing standard of care were noted in this limited cohort of patients. Future attempts to utilize checkpoint inhibitor immunotherapy in the neoadjuvant setting should consider focusing on novel methods of enhancing response rates and addressing micro-metastatic disease. 
